# Antibiotic prophylaxis for oral lacerations: our emergency department’s experience

**DOI:** 10.1186/s12245-016-0122-7

**Published:** 2016-09-13

**Authors:** Suzanne Lilley Katsetos, Roxanne Nagurka, Jaclyn Caffrey, Steven E. Keller, Tiffany Murano

**Affiliations:** Department of Emergency Medicine, Rutgers New Jersey Medical School, 150 Bergen Street, M203, Newark, NJ 07101 USA

**Keywords:** Emergency department, Oral lacerations, Antibiotic prophylaxis

## Abstract

**Background:**

The purpose of this study was to examine the emergency physician (EP) practice of prescribing prophylactic antibiotics for patients with oral lacerations. A secondary outcome measure was the infection rate of those who were or were not prescribed antibiotics.

**Methods:**

The study was a retrospective chart review of 323 patients who presented to a large urban emergency department (ED) between January 1, 2012 and December 31, 2012 with an oral laceration.

**Results:**

Of the 323 charts reviewed, topical and/or systemic antibiotics were prescribed in the ED to 62 % (199/323) of patients. Of those patients, 38 % (75/199) received only topical antibiotics, 34 % (68/199) received only systemic antibiotics, and 28 % (56/199) were prescribed topical and systemic antibiotics. Thirty-eight percent (124/323) of patients received no antibiotics. Eighteen percent (58/323) of patients returned for follow-up with an infection rate of 10 % (6/58). There was a statistical difference in rates of infection between patients who received antibiotics and who did not receive antibiotics and a statistical difference in rates of infection between patients with complex lacerations who received and did not receive antibiotic.

**Conclusions:**

This study shows that there is a considerable amount of practice variance in prescribing prophylactic antibiotics for oral lacerations among EPs in our ED. Due to the poor follow-up rate, an accurate infection rate could not be determined. In the future, adequately powered randomized controlled studies may provide compelling data for or against the necessity for prophylactic antibiotic use for oral lacerations.

## Background

Lacerations of the oral cavity are injuries that commonly present to the emergency department (ED) [1]. The oral cavity is bathed in bacteria-rich secretions, as well as food and other foreign material. Previous studies have reported infection rates for lacerations to the oral cavity (including tongue, lips, and mucosa) between 4.3 and 23.1 % [2–5], which is higher than the overall infection rate of 7.2 % for traumatic wounds [6].

It seems logical that this high-risk environment would necessitate the use of prophylactic antibiotics. However, there is no compelling data that demonstrates the effectiveness or identifies a specific patient population who would benefit from antibiotic use for preventing infection in oral lacerations. Most of the studies to date that have attempted to examine the use of prophylactic antibiotics in patients with oral lacerations have been inadequately powered leading to inconclusive results [1–6].

One emergency medicine (EM) textbook recommends a prophylactic course of antibiotics for large mucosal wounds (1–2 cm) and those that are through-and-through wounds [7]. However, another states that rigorous oral hygiene and intraoral rinses are sufficient due to the lack of evidence supporting prophylactic antibiotics in patients with sutured oral lacerations [8].

We hypothesized that this lack of compelling research has led to practice variation by emergency physicians (EP). The purpose of this study was to examine the EP practice of prescribing prophylactic antibiotics for patients who have oral lacerations in an ED. A secondary outcome measure was the infection rate of those who were and were not prescribed antibiotics.

## Methods

This study was a retrospective chart review of patients who presented to an academic-based urban ED in Newark, NJ, with approximately 100,000 annual visits. The electronic medical record (EMR) database was queried for patients who had a discharge diagnosis of “open wound of lip, tongue, mouth, gum, face, buccal mucosa, or palate” between January 1, 2012 and December 31, 2012. Patients were excluded if they did not have an oral laceration, if they had a pre-existing infection, were already taking antibiotics, had a laceration from human or animal bite, or had a laceration that occurred greater than 12 h prior to presentation to the ED. Data were collected from the ED EMR and oral surgery clinic medical records.

Five research assistants with the use of a detailed codebook collected data. One blinded individual answered discrepancies. Data points collected included: type of laceration, location (tongue, oral mucosa, or lip), complexity (simple or complex), use of sutures (suture repair or no suture repair), suture material (absorbable or non-absorbable), use of antibiotics (topical, systemic or both), and status of wound at return visit (infected or not infected). Demographic data such as age, gender, immune status, and mechanism of injury were also obtained.

Lacerations were classified as complex if they were through and through or greater than 2 cm. All other lacerations were classified as simple. Patients were defined as immunocompromised if they had HIV or diabetes, were taking glucocorticoid therapy or post-transplant immunosuppressants, or were undergoing chemotherapy or radiation. Criteria for infection included any documentation in the chart of “drainage or discharge, erythema, redness, swelling, cellulitis, abscess, or fever.”

Institutional Review Board approval was obtained prior to data collection. Microsoft Excel was used for data tabulation, and IBM SPSS Statistics was used for statistical analysis. A one-tailed *t* test was used to compare infection rates. We compared two independent proportions using z-ratios and exact tests; it is of note that two cells had small *n* and that larger sample sizes are required to increase confidence in the *P* values.

## Results

A total of 323 patients met inclusion criteria. Patients ranged in age from 10 months to 96 years of age. The average age of patients was 26 years of age, and the median age was 24 years (see Table 1). The majority of the patients were male (69 %; 222/323) and 31 % (101/323) were female. Four percent of the patients (14/323) were immunocompromised; four patients had HIV, eight patients had diabetes, one was currently undergoing chemotherapy, and one was on systemic steroids.

**Table 1:** Demographic patient data

	Male	Female	Total
*N* (%)	222 (69)	101 (31)	323
Mean age (years)	26	25	26
Median age (years)	24	21	24

All of the presenting injuries were the result of a traumatic mechanism. Eighty-one percent (262/323) of patients had an isolated oral laceration with no other injury. Seventy-seven percent (250/323) had lip lacerations, 24 % (78/323) had oral mucosa lacerations, and 10 % (32/323) had tongue lacerations (see Table 2).

**Table 2:** Wound location, complexity, and repair technique

Wound location	*N* (%)					
	Total	Simple	Complex	Sutured		
				Total	Absorbable	Non-absorbable
Lip	250 (77)	161 (64)	89 (36)	144 (58)	59 (41)	85 (59)
Mucosa	78 (24)	58 (74)	20 (26)	36 (46)	30 (83)	6 (17)
Tongue	32 (10)	25 (78)	7 (22)	7 (22)	7 (100)	0 (0)

Lacerations were recorded as simple in the following locations: 64 % (161/250) in the lip, 74 % (58/78) in the oral mucosa, and 78 % (25/32) in the tongue. Complex lacerations were found in 36 % (89/250) of the lip, 26 % (20/78) of the oral mucosa, and 22 % (7/32) of the tongue (see Table 2).

Fifty-three percent (170/323) of patients had sutures placed to at least one wound location and 47 % (153/323) had no sutures placed. Sutures were used for wound repair in 58 % (144/250) of lip lacerations, 46 % (36/78) of oral mucosa, and 22 % (7/32) of tongue lacerations. Absorbable sutures were used in 41 % (59/144) of lip lacerations, 83 % (30/36) of oral mucosa lacerations, and 100 % (7/7) of tongue wounds. Non-absorbable sutures were used in 59 % (85/144) of lip lacerations, 17 % (6/36) of oral mucosa lacerations, and 0 % (0/7) of tongue wounds (see Table 2).

Of the 323 charts reviewed, topical and/or systemic antibiotics were prescribed in the ED to 62 % (199/323) of patients (see Table 3). Of those patients, 38 % (75/199) received only topical antibiotics, 34 % (68/199) received only systemic antibiotics, and 28 % (56/199) were prescribed topical and systemic antibiotics (see Table 4). Thirty-eight percent (124/323) of patients received no antibiotics. Prescribed topical antibiotics included bacitracin zinc ointment and systemic antibiotics included penicillin V potassium, clindamycin, cephalexin, amoxicillin, augmentin, and ciprofloxacin.

**Table 3:** Patients prescribed antibiotics

	*N* (%)
Prescribed antibiotics	199 (62)
Not prescribed antibiotics	124 (38)

**Table 4:** Type of antibiotic prescribed

	*N* (%)
Topical only	75 (38)
Systemic only	68 (34)
Both topical and systemic	56 (28)

Eighteen percent (58/323) of patients returned for follow-up wound care either to the ED or the oral surgery clinic. Of those who returned, 40 patients (69 %) had been prescribed antibiotics and 18 patients (31 %) had not been prescribed antibiotics. Of those 58 patients who returned, 10 % (6/58) had an infection. One of the six patients who had an infection had been prescribed systemic antibiotics, and one had been prescribed systemic and topical antibiotics. The remaining four patients had not been initially prescribed antibiotics (see Table 5). Therefore, the ratio of infection in patients prescribed antibiotics was 2/40 (5 %), and the ratio of infection in patients not prescribed antibiotics was 4/18 (22 %). Statistical analysis revealed a significant difference between these two independent proportions (one-tailed *p* < 0.023, see Table 6).

**Table 5:** Infection status of patients who returned for follow-up wound care

	*N* (%)		
	Infected	Not infected	Total
Topical antibiotics	0 (0)	11 (100)	11
Systemic antibiotics	1 (6)	16 (94)	17
Both topical and systemic antibiotics	1 (8)	11 (92)	12
No antibiotics prescribed	4 (22)	14 (78)	18
Total	6 (10)	52 (90)	58

**Table 6:** One-tailed *p* values for patients who returned

	Rate of infection (%)		
	+Abx	−Abx	*P* value
Patients who returned	2/40 (0.05)	4/18 (0.22)	0.023
Patients who returned excluding immunocompromised	2/38 (0.53)	3/17 (0.18)	0.069
Simple lacerations (no IC patients)	0/21 (0)	1/10 (0.1)	0.071
Complex lacerations	2/19 (0.11)	3/8 (0.38)	0.049
Complex lacerations excluding immunocompromised	2/19 (0.11)	2/7 (0.29)	0.129

Of the 58 patients who returned, three were immunocompromised. Two of the patients had been prescribed antibiotics, and one had not been prescribed antibiotics. Both of the immunocompromised patients who were initially prescribed antibiotics had simple lacerations and did not develop an infection. The immunocompromised patient who developed an infection had not been prescribed antibiotics and had a complex laceration. When excluding immunocompromised patients, the ratio of infection in patients prescribed antibiotics was 2/38 (5 %), and the ratio of infection in patients not prescribed antibiotics was 3/17 (18 %). Statistical analysis revealed no statistical difference between the rates of infection (one-tailed *p* < 0.069; see Table 6).

In addition, the ratio of infection in patients with simple lacerations prescribed antibiotics was 0/21, and the ratio of infection in patients with simple lacerations not prescribed antibiotics was 1/10. Therefore, no immunocompromised patients were included. There was no statistical difference the ratio of infection in patients with simple lacerations who were prescribed antibiotics versus patients with simple lacerations who were not prescribed antibiotics (*p* < 0.071).

Similarly, the ratio of infection in patients with complex lacerations prescribed antibiotics was 2/19 (10 %), and the ratio of infection in patients with complex lacerations not prescribed antibiotics was 3/8 (37 %). There was a statistically significant difference in the rates of infection in complex lacerations with or without antibiotic use (one-tailed *p* < 0.049). However, when excluding immunocompromised patients, there is no statistical difference the ratio of infection in patients with complex lacerations who were prescribed antibiotics (2/19; 10 %) versus patients with complex lacerations who were not prescribed antibiotics [(2/7; 28 %) *p* < 0.129].

There was no common type of laceration or suture material in the six patients who returned with an infection. One patient did not require sutures for his lip laceration. Two patients received non-absorbable and absorbable sutures to a lip laceration, and one patient had non-absorbable sutures placed in a lip laceration. Two patients received non-absorbable sutures in lip and oral mucosa.

## Discussion

This study found that there is great variability in treating oral lacerations with antibiotics. No antibiotics were prescribed for 38 % (124/323) of patients. Of the 199 patients who were prescribed antibiotics, 38 % (75/199) received only topical antibiotics, 34 % (68/199) received only systemic antibiotics, and 28 % (56/199) received both topical and systemic antibiotics. This lack of consistency between treating EPs is consistent with the lack of research and conflicting expert recommendations.

Topical antibiotics (bacitracin zinc ointment) were prescribed to 66 % of patients (131/199). This practice is consistent with previous documented use of topical antibiotics for wound care. In a national survey of wound management, Howell and Chisholm found that 71 % of respondents used gauze and a topical antibiotic for wound care of simple lacerations [9]. This is an interesting finding as there is a paucity of evidence for use of topical antibiotics in uncomplicated traumatic lacerations to prevent infection. Dire et al. performed a prospective double-blind randomized placebo-controlled study comparing infection rates of four common topical antibiotic ointments versus placebo in minor repaired wounds. The authors found that the infection rate was 5.5 % for bacitracin and 12.2 % in the placebo group. However, that study did not include immunocompromised patients [10]. More recently, published meta-analysis of prophylactic topical antibiotic use for uncomplicated soft tissue wounds found only four studies addressing this issue [11]. Based on these studies, the authors concluded that topical antibiotics reduce the infection rate of minor uncomplicated soft-tissue wounds. The authors also found that the placebo arms of these studies had an overall higher infection rate above that previously reported in general ED populations and concluded that topical preparations that increase the moisture, but do not include an antibiotic have an increased infection rate and therefore do not recommend its use. Our study revealed that patients who received only topical antibiotics had an infection rate of zero. However, the overall infection rate was so low in both this group and the no antibiotic use group that it cannot be inferred that there is a distinct benefit from its use.

The overall infection rate in patients who returned (10 %) is consistent with previous infection rates for oral lacerations found in previous studies [2–5]. However, the majority of patients were lost to follow-up as they did not return following the initial ED visit. This may be due to the fact that 47 % (153/323) of patients did not have any sutures placed, and in the wounds that were sutured, the majority of the wounds were repaired with absorbable sutures (41 % of lip lacerations, 83 % of oral mucosa lacerations, and 100 % of tongue wounds) which did not require that patients return to the ED for removal or intervention.

The rate of sutures placed in the 58 patients who returned was 72 % (42/58), suggesting that patients were more likely to return if they had sutures placed. Discharge instructions were given to patients to return with any signs of infection. If one extrapolates that the patients who did not follow-up did not have an infection, then the infection rate was 2 % (6/323). This situation is similar to that experienced by Lammers et al. who prospectively studied predictive factors in uncomplicated traumatic wounds in the ED over a 3 year period. The follow-up rate in that study was 24 % with an overall infection rate of 7.2 % [6]. When they assumed that patients who failed to return did not have infections, their overall infection rate was 1.8 %. It is important to note however, that Lammers study excluded patients with intra-oral lacerations [6].

There are several important factors to be considered when risk stratifying wounds that are more likely to become infected and therefore would benefit from prophylactic antibiotics. One risk factor for wound infection is immunocompromised host status, which includes diabetes mellitus, HIV disease, and treatment with such agents as corticosteroids and chemotherapeutics. One study by Hollander et al. found that although these medical conditions (specifically diabetes mellitus) comprised less than 0.5 % of their study population, these patients had a 3.9 relative risk for infection [12]. In our study, there were 14/323 patients (4 %) who were immunocompromised. Only one of the three immunocompromised patients who returned for follow-up had an infection, and this was in complex laceration that was not initially prescribed antibiotics. The other two immunocompromised patients had simple lacerations, had been prescribed antibiotics, and did not have an infection upon return visit.

Another factor to consider when risk stratifying wounds is the complexity of the wound. In our study, there was a statistically significant difference between the ratios of infection in patients with complex lacerations prescribed versus not prescribed antibiotics. Similarly, Steele et al. randomized 62 adult patients presenting within 24 h of injury with full-thickness intraoral lacerations to either treatment with pen VK for 5 days or placebo therapy. In a subgroup analysis of full-thickness lacerations, 7 % (1/14) versus 27 % (4/15) of patients developed wound infection in the treatment and placebo groups, respectively (RR = 0.27, 95 % CI = 0.03 to 2.12) [5]. Steele concluded that patients with full-thickness lacerations may benefit proportionally more from prophylaxis than simple lacerations, but reported an inconclusive analysis due to an underpowered clinical trial.

Lastly, there was a statistical difference in rates of infection between patients who received antibiotics and who did not receive antibiotics and a statistical difference in rates of infection between patients with complex lacerations who received and did not receive antibiotic. However, when immunocompromised patients were excluded, there was no statistical difference in either group.

### Limitations

One of the main limitations of our study is the low return rate, which limits the power of our study. Our assumption is that patients did not return if they did not have any concerns about infection. Another explanation may be that patients did not return if they did not have wounds that were sutured or if they had absorbable sutures. However, only 30/91 patients (33 %) with non-absorbable sutures returned for follow-up and removal. There was a large number of patients with non-absorbable sutures that are unaccounted for, and it is unknown how their sutures were removed or if they visited another physician. In addition, due to the retrospective nature of the study, not only were the antibiotic types, dosage, and duration non-standardized but also we were unable to ascertain the compliance rate to prescribed antibiotics. The infection rate was also low which may limit the power and confidence in statistical comparisons. Finally, although an extensive codebook was used and coders were educated before coding began, certain discrepancies in data are concerning. For example, non-absorbable sutures were used in 17 % of oral mucosa lacerations. This seems high given that most EPs would use absorbable sutures in mucosa and raises questions about inter-coder reliability.

## Conclusions

This study shows that there is a considerable amount of practice variance in prescribing prophylactic antibiotics for oral lacerations among EPs in our ED. The inconsistency may be due to inadequate supporting data. Because of the poor follow-up rate, the infection rate could not be accurately determined; however our data suggests that immunocompromised patients may benefit from prophylactic antibiotics. In the future, adequately powered randomized controlled studies may provide compelling data for or against the necessity for prophylactic antibiotic use for oral lacerations.
